# Weight Loss Outcomes With Telemedicine During COVID-19

**DOI:** 10.3389/fendo.2022.793290

**Published:** 2022-03-10

**Authors:** Beverly G. Tchang, Chenel Morrison, Joon Tae Kim, Farheen Ahmed, Karina M. Chan, Laura C. Alonso, Louis J. Aronne, Alpana P. Shukla

**Affiliations:** ^1^ Department of Medicine, Division of Endocrinology, Diabetes and Metabolism, Comprehensive Weight Control Center, Weill Cornell Medicine, New York, NY, United States; ^2^ Weill Cornell Medical College, New York, NY, United States; ^3^ Weill Cornell Medical College-Qatar, Ar-Rayyan, Qatar; ^4^ Institute of Human Nutrition, Columbia University, New York, NY, United States; ^5^ Department of Medicine, Division of Endocrinology, Diabetes and Metabolism, Weill Cornell Medicine, New York, NY, United States

**Keywords:** obesity, weight, telemedicine, telehealth, COVID-19, SARS-CoV-2, anti-obesity medication, video

## Abstract

**Background:**

Amidst the COVID-19 pandemic, telemedicine was rapidly implemented to maintain patient care during quarantine. However, there is little data on how this transition may have impacted weight loss outcomes and interventions among patients with overweight or obesity.

**Methods:**

This was a retrospective observational study of adults who established care for medically managed obesity at the Weill Cornell Comprehensive Weight Control Center during September-November 2019 and May-July 2020 and who completed 6 months of follow-up. Weight loss outcomes and weight management interventions were explored and stratified by patient-provider interaction: in-person visits only, in-person and video visits, and video visits only.

**Results:**

Of 499 charts eligible for review, 245 (49%) returned for their 6-month follow-up visit and were included for analysis. Of 245 patients, 69 had in-person visits only (“in-person”), 85 started in-person and later switched to video visits (“hybrid”), and 91 had video visits only (“video”). All cohorts were predominantly white and female. Median ages were 56, 49, and 49 years; baseline median weights were 98.9, 96.8, and 93.0 kg; and baseline median BMIs were 35.3, 34.4, and 34.0 kg/m^2^ for in-person, hybrid, and video cohorts, respectively. The median percent weight changes over 6 months were not significantly different among cohorts: -4.3% [-8.5, -1.5] in the in-person cohort, -5.6% [-8.7, -2.2] in the hybrid group, and -5.8% [-9.7, -2.4] in the video cohort. The percent of patients who achieved ≥5% weight loss were also similar: 46.4%, 55.3%, and 59.3%, respectively. The median number of visits in the video cohort was more than in the in-person or hybrid groups (5 vs. 4). Median number of anti-obesity medications (AOMs) prescribed was similar among groups. The most common AOMs were metformin (all cohorts) followed by semaglutide 1.0 mg (in-person and video) or topiramate (hybrid).

**Conclusion:**

Patients on anti-obesity medications who were followed for 6 months *via* video or video plus in-person visits (hybrid) experienced clinically significant weight loss. Median number of AOMs were similar among groups, and the most common AOMs were metformin, semaglutide 1.0 mg, and topiramate. More investigation is required to compare telemedicine models with in-person care.

## Introduction

The epidemic of obesity in the United States contributes to significant morbidity and mortality as well as a financial burden to both patients and the public. Between 2000-2017, the prevalence of obesity in the U.S. increased from 30.5% to 43.4% (1). Obesity contributes to the development of heart disease, stroke, type 2 diabetes (T2D), and cancer, which are among the leading causes of preventable and premature death (2). Obesity also increases annual medical expenditures financed by the public sector by 7-11% (3).

The COVID-19 pandemic further exacerbated these personal and public health burdens associated with obesity. With mandatory stay-at-home orders, patients reported increased anxiety and depression and were less likely to achieve their weight loss goals (4). Access to care shifted primarily to telemedicine, which may or may not be comparable to in-person visits depending on the modality (e.g., web, telephone, email, text, or smartphone app) (5).

Comparisons between video visits and in-person visits have been limited to lifestyle interventions only and vary in their methods and populations (6–10). A retrospective study of 100 adults in Peru found no significant differences in weight loss between telenutrition vs. in-person counseling (10). In a prospective study of 1550 participants in France who received nutrition and behavioral counseling, less weight loss was observed during the COVID-19 lockdown than before the lockdown (9). Behavioral intervention trials prior to COVID-19 found similar (6, 7) or more weight loss (8) when videoconferencing was compared to in-person delivery. However, these studies were conducted in participants undergoing lifestyle interventions alone. Studies in medically managed obesity evaluated patients’ or providers’ perspectives on the use of telemedicine but did not report weight loss outcomes (11, 12). There are no studies that have investigated the relationship between video visits and weight loss outcomes in patients with overweight/obesity treated with anti-obesity pharmacotherapy. The COVID-19 pandemic and quarantine policies provided a unique socio-ecological framework to observe clinical outcomes and management practices over a telemedicine transition.

In this retrospective observational study, we explored weight loss outcomes and weight management interventions among three populations of patients with medically managed overweight/obesity who received care *via* in-person visits only, in-person and video visits, and video visits only.

## Methods

This is a retrospective observational study of new patients who established care for overweight or obesity (BMI ≥ 25 kg/m^2^) from September 2019 through November 2019 or May 2020 through July 2020 at the Weill Cornell Comprehensive Weight Control Center in New York. Time periods were selected to represent cohorts prior to and after lockdown implementation. During these time periods, the Weill Cornell Comprehensive Weight Control Center employed ten physicians (four endocrinologists), one nurse practitioner, and three registered dietitians. Patients who attended in-person visits were weighed in-person; those who attended video visits provided self-reported weights from home scales or other medical office visits. Primary outcomes of interest were percent weight loss from initial visit, proportion of patients who achieved clinically significant weight loss defined as ≥ 5% weight loss (2), and proportion of patients who achieved ≥ 10% weight loss. Secondary outcomes included number of physician or dietitian visits and the number and types of anti-obesity medications (AOMs) prescribed. AOMs were defined as any medication prescribed on- or off-label for weight loss.

Data was abstracted through electronic medical query, and manual chart review was used to extract and confirm data pertaining to demographics, medical history, medications, and weight changes. The database was stored and managed with the Research Electronic Data Capture (REDCap) tool hosted at Weill Cornell Medicine. All patients who established care during one of the defined timeframes and returned for an in-person or video follow-up visit within 6 ± 3 months were included. Patients were excluded if they presented for a non-weight-related chief complaint, had a history of bariatric surgery, were pregnant, had telephone appointments, or were missing relevant data (e.g., height).

### Statistical Analysis

Data were analyzed in R Core Team (2020) (13), using standard R software functions (summary, aov, lm, estimable, dredge, ggplot, etc.) invoked by the *cufunctions* package (14). Variables were compared among the three cohorts using Kruskal-Wallis rank sum test due to a non-normative distribution. Categorical variables were compared using Fisher’s exact p-value or Pearson’s Chi-squared test.

## Results

Of 541 charts identified by automated electronic medical record query, 25 were removed due to ineligibility and 17 were removed for incomplete data, resulting in 499 charts eligible for review (**Supplemental Figure 1**). After excluding 254 due to lack of a follow-up visit in the prespecified time frame, 245 (49%) records were analyzed. Of 245 patients, 69 had in-person visits only (“in-person”), 85 had in-person and video visits (“hybrid”), and 91 had video visits only (“video”). All cohorts were predominantly white and female (**Table 1**). Median ages were 56, 49, and 49 years, baseline median weights were 98.9, 96.8, and 93.0 kg, and baseline median BMIs were 35.3, 34.4, and 34.0 kg/m^2^ for in-person, hybrid, and video cohorts, respectively. The most common comorbidities among all groups were hyperlipidemia followed by hypertension or prediabetes. The prevalence of T2D was 9%, 15%, and 8% in the in-person, hybrid, and video cohorts, respectively. Baseline characteristics were similar among all groups except for age and prevalence of non-alcoholic fatty liver disease and eating disorders.

**Table 1:** Baseline characteristics of in-person, hybrid, and video cohorts.

	In-person (n = 69)	Hybrid (n = 85)	Video (n = 91)	p-value
Median age, years	56 [44, 62]	49 [38, 57]	49 [37, 60]	**0.026**
Female	50 (72)	64 (75)	71 (78)	0.72
Weight, kg	98.9 [86.8, 113.5]	96.8 [84.5, 112.4]	93.0 [82.2, 111.3]	0.52
BMI, kg/m^2^	35.3 [32.3, 39.1]	34.4 [31.6, 41.2]	34.0 [30.7, 37.7]	0.24
AOMs, prior to initial visit^§^	1 [1, 1]	1 [1, 1]	1 [1, 1]	0.52
Most common AOM, prior to initial visit	Metformin 18 (26)	Metformin 18 (21)	Metformin 17 (19)	0.53
Race				0.15
White	33 (75)	44 (70)	53 (75)	
Black	5 (11)	5 (8)	12 (17)	
Asian	0 (0)	5 (8)	2 (3)	
Native Hawaiian/Pacific Islander	1 (2)	0 (0)	0 (0)	
Other	5 (11)	9 (14)	4 (6)	
Not reported	25 (38)	22 (29)	20 (22)	
Smoking				0.28
Current	4 (6)	3 (4)	3 (3)	
Former	13 (19)	8 (9)	17 (19)	
Never	42 (61)	62 (73)	45 (49)	
Not reported	10 (15)	12 (14)	26 (29)	
Comorbidities				
T2D	6 (9)	13 (15)	7 (8)	0.25
Prediabetes	30 (43)	29 (34)	35 (38)	0.49
HLD	51 (74)	59 (69)	55 (60)	0.17
HTN	30 (43)	24 (28)	23 (25)	0.56
CVD	5 (7)	3 (4)	4 (4)	0.56
NAFLD	5 (7)	9 (11)	2 (2)	**0.049**
OSA	10 (14)	19 (22)	12 (13)	0.22
PCOS	4 (6)	9 (11)	10 (11)	0.53
Depression	15 (22)	18 (21)	14 (15)	0.51
Anxiety	10 (14)	15 (18)	12 (13)	0.70
Eating disorder*	14 (20)	15 (19)	6 (7)	**0.018**

Statistics presented as median [interquartile range] or n (%). *Eating disorders included history of anorexia nervosa, bulimia, or binge eating. ^§^Anti-obesity medications included any medication prescribed on- or off-label for weight loss. AOMs, anti-obesity medications; BMI, body mass index; CVD, cardiovascular disease; HLD, hyperlipidemia or dyslipidemia; HTN, hypertension; NAFLD, non-alcohol fatty liver disease; OSA, obstructive sleep apnea; PCOS, polycystic ovarian syndrome; T2D, type 2 diabetes.

Bolded values indicate statistical significance, p<0.05.

**Figure 1:**
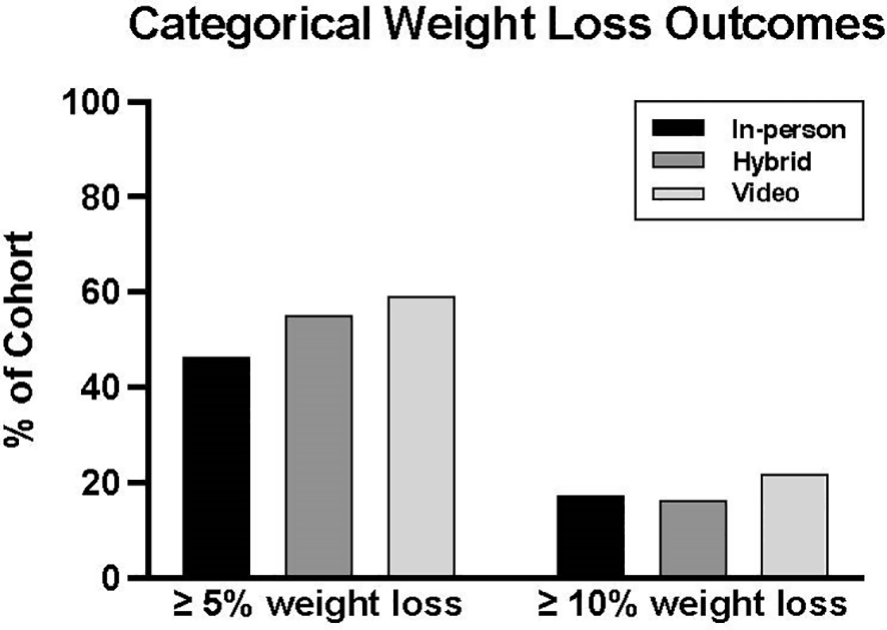
Categorical weight loss outcomes in in-person, hybrid, and video cohorts.

Weight loss outcomes were not significantly different between groups. The median percent weight changes were -4.3% [-8.5, -1.5] in the in-person cohort, -5.6% [-8.7, -2.2] in the hybrid group, and -5.8% [-9.7, -2.4] in the video cohort (**Table 2**). The percentages of patients who experienced ≥5% and ≥10% weight loss were also similar: 46.4%, 55.3%, and 59.3% achieved ≥5% weight loss, and 17.4%, 16.5%, and 22.0% achieved ≥10% weight loss in the in-person, hybrid, and video groups, respectively (**Figure 1**).

**Table 2:** Weight loss outcomes among in-person, hybrid, and video cohorts.

	In-person	Hybrid	Video	p-value
Weight change (%)	-4.3 [-8.5, -1.5]	-5.6 [-8.7, -2.2]	-5.8 [-9.7, -2.4]	0.41
BMI change (kg/m^2^)	-1.4 [-3.2, -0.6]	-2.0 [-3.3, -0.8]	-2.1 [-3.6, -0.7]	0.58
≥5% weight loss	32 (46.4)	47 (55.3)	54 (59.3)	0.26
≥10% weight loss	12 (17.4)	14 (16.5)	20 (22.0)	0.61

Statistics presented as median [interquartile range] or n (%). BMI, body mass index.

The total number of visits were significantly different among the cohorts, with 5 visits in the video group and 4 visits each in the hybrid and in-person groups, which was driven by significantly more visits with providers (physician or nurse practitioner) (**Table 3**). In-person, video, and hybrid cohorts had a median of 3 [2,3], 3 [3,4] and 4 [3,5] provider visits, respectively. The number of visits with a registered dietitian was not significantly different among groups. Patients’ 6-month weights were observed at a significantly earlier time point in the in-person group (130 days) than in the hybrid group (185 days) and video group (179 days). The most common AOMs prescribed at the initial visit were metformin followed by semaglutide 1.0 mg or topiramate. These medications remained the most common at 6 months.

**Table 3:** Secondary outcomes among in-person, hybrid, and video cohorts.

	In-person	Hybrid	Video	p-value
All visits	4 [3, 4]	4 [3, 6]	5 [4, 7]	**<0.05**
MD/NP visits	3 [2, 3]	3 [3, 4]	4 [3, 5]	**<0.05**
RD visits	1 [0, 2]	0 [0, 2]	1 [0, 3]	0.56
Days to 6-month appointment	130 [108, 156]	185 [163, 216]	179 [140, 210]	**<0.05**
AOMs at 6 months	1 [1, 2]	2 [1, 2]	2 [1, 2]	0.089
Most common AOM, initial^‡^	Metformin 53 (77)	Metformin 56 (66)	Metformin 60 (66)	
2^nd^ most common AOM, initial^‡^	Semaglutide 1.0 mg 10 (14)	Topiramate 11 (13)	Semaglutide 1.0 mg 10 (11)	
Most common AOM, 6 months	Metformin 58 (83)	Metformin 59 (70)	Metformin 69 (76)	
2^nd^ most common AOM, 6 months	Semaglutide 1.0 mg 15 (21)	Topiramate 20 (24)	Semaglutide 1.0 mg 17 (19)	

^‡^AOMs that were newly prescribed at the initial visit. Statistics presented as median [interquartile range] or n (%). AOM, anti-obesity medication; MD/NP, physician/nurse practitioner; RD, registered dietitian.

Bolded values indicate statistical significance, p < 0.05.

## Discussion

This retrospective observational study is the first to describe weight loss outcomes in patients with medically managed obesity across a telemedicine transition, represented by three cohorts of patients who were followed for 6-months with in-person visits only, in-person and video visits, or video visits only. Percent weight loss as well as ≥5% and ≥10% categorical weight loss outcomes were not significantly different among the groups. Though this investigation was hindered by small sample size, similar outcomes have been reported in a small randomized controlled trial (6) and a large intervention trial (15) that evaluated the effect of lifestyle counseling administered *via* in-person versus video visits.

The telemedicine cohort had more visits than the in-person and hybrid cohorts. Higher frequency of engagements with providers counseling on lifestyle interventions predicts greater weight loss (2), and telemedicine may be a viable strategy to support more frequent patient-provider interaction by removing barriers to care (e.g., travel time, time off from work) (16). We previously reported a 27.2% reduction in our no-show rate that may be reflective of this effect (17), a trend that has also been described in other medical specialties (18).

With the benefits of long-term safety data and low cost, metformin was the most commonly prescribed AOM. Durable weight loss with metformin was observed out to 15 years in the Diabetes Prevention Program (19), and we previously reported real-world data of 6-8% weight loss over one year of metformin therapy among individuals with normoglycemia, prediabetes, or T2D (20). Currently, the most efficacious AOM is semaglutide 2.4 mg (21–24), which was approved in June 2021. Because semaglutide 2.4 mg was not yet approved at the time of this study, it was unsurprising that the second most common AOM prescribed in two of our three cohorts was semaglutide 1.0 mg, which has demonstrated weight loss efficacy as a secondary endpoint in phase 3 trials of patients with T2D (25, 26). Topiramate was the second most common AOM prescribed in our hybrid cohort, whose follow-up spanned the period early in the COVID-19 pandemic, during which our patients self-reported more cravings and comfort eating (data not published). Given the proven weight loss efficacy of semaglutide in RCTs, it was surprising that the in-person and video groups did not lose more weight than the hybrid group. This lack of observed difference likely reflects a combination of clinical factors (e.g., fewer MD/NP visits with in-person cohort) and methodological factors (e.g., small sample sizes).

In our study, both metformin and semaglutide 1.0 mg were used off-label for the treatment of obesity. Off-label prescribing practices are often necessitated by the lack of insurance coverage for obesity pharmacotherapy (27). Our utilization of semaglutide 1.0 mg as an off-label AOM may reflect adequate insurance coverage for obesity-related comorbidities such prediabetes, which represented about a third of our cohorts.

This exploratory study is strengthened by the inclusion of both on- and off-label AOMs, granularity in prescribing practices (i.e., AOMs present prior to first visit, initial AOMs prescribed, and AOMs ultimately used at 6 months), follow-up to 6 months, and the addition of a hybrid group that highlighted the transition to telemedicine. Limitations include a small sample size, predominantly white population, and self-reported weights in the hybrid and video cohorts. Because this was an observational study, we were able to draw only hypothesis-generating conclusions. Some data of interest were unavailable (e.g., smoking status), and cohorts were not matched. Eating disorder prevalence, for example, was higher in the in-person and hybrid groups than the video group, which may allude to a barrier to disclose or elicit an eating disorder history. The different prevalence of eating disorders in the three groups may have impacted observations. Another comorbidity potentially unbalanced among our cohorts was OSA. The prevalence of OSA was numerically but not statistically higher in the hybrid group (n=19) than in the in-person (n=10) or video groups (n=12). The effect of OSA on weight loss is debated. Impaired sleep has been associated with increased levels of the orexigenic hormone ghrelin, which may impair weight loss efforts (28). However, an increase in basal metabolic rate because of frequent nighttime awakenings has also been observed in OSA (29). Because the pathophysiology linking obesity and OSA is unclear, the higher prevalence of OSA in the hybrid group may have impaired or benefited their weight loss efforts. Furthermore, a meta-analysis of RCTs found that treatment of OSA was associated with slight weight gain, but data regarding treatment (e.g., untreated, CPAP or oral device, adherence) was unavailable for this study (30). Future studies should assess the efficacy of telemedicine over a longer observation period, investigate outcomes in different ethnic populations, assess body composition changes (e.g., fat mass, lean body mass), and incorporate remote weight monitoring.

The widespread adoption of telemedicine necessitated by the COVID-19 pandemic is propelling a paradigm shift in the delivery of patient care. Telehealth has been proven to be feasible and effective in multiple fields including primary care, pediatrics, endocrinology, geriatrics, and psychiatry (31–35). Our exploratory data suggest that telemedicine was similarly effective to in person visits for weight loss outcomes over this time frame. In the field of obesity medicine, telehealth might be a strategy to reach specific racial/ethnic populations who are at higher risk of obesity (36, 37). Further studies are needed to guide health professionals in the use of video visits as part of obesity management.

## Data Availability Statement

The data analyzed in this study is subject to the following licenses/restrictions: Dataset was generated through electronic medical record query and is stored on a HIPAA-secure system that contains patient information, which cannot be shared outside of those authorized as research staff per IRB protocol. Requests to access these datasets should be directed to bgt9001@med.cornell.edu.

## Ethics Statement

The studies involving human participants were reviewed and approved by Weill Cornell Medicine Institutional Review Board (IRB 21-02023263). Written informed consent for participation was not required for this study in accordance with the national legislation and the institutional requirements.

## Author Contributions

BT and AS were responsible for study concept and design. Data acquisition was performed by CM, JK, and FA. Data analysis was performed by CM, JK, FA, and KC. All authors were involved in data interpretation. Manuscript was prepared by BT, CM, FA, KC and JK. All authors participated in manuscript review and edits. Study was supervised by BT and AS. All authors contributed to the article and approved the submitted version.

## Funding

CM was supported by the Dr. Robert C. and Veronica Atkins Foundation Curriculum in Metabolic Diseases Scholarship.

## Conflict of Interest

LJA reports receiving consulting fees from and serving on advisory boards for Jamieson Laboratories, Pfizer, Novo Nordisk, Eisai, Erx Pharmaceuticals, Real Appeal, Janssen Pharmaceuticals, and Gelesis; receiving research funding from Aspire Bariatrics, Allurion, Eisai, AstraZeneca, Gelesis, Janssen Pharmaceuticals and Novo Nordisk; having equity interests in Intellihealth Corp, Allurion, Erx Pharmaceuticals, Zafgen, Gelesis, Myos Corp., and Jamieson Laboratories; and serving on a board of directors for Intellihealth Corp., Myos Corp., and Jamieson Laboratories. BGT reports consulting and/or commission fees from Novo Nordisk, 2nd.MD, Intellihealth, and Elsevier.

The remaining authors declare that the research was conducted in the absence of any commercial or financial relationships that could be construed as a potential conflict of interest.

## Publisher’s Note

All claims expressed in this article are solely those of the authors and do not necessarily represent those of their affiliated organizations, or those of the publisher, the editors and the reviewers. Any product that may be evaluated in this article, or claim that may be made by its manufacturer, is not guaranteed or endorsed by the publisher.

## Supplementary Material

Supplementary Figure 1 Flowchart of patients included for analysis.
