# Supplementary figures and images for: Weight Loss Outcomes With Telemedicine During COVID-19
Source: Front Endocrinol (Lausanne). 2022 Mar 10;13:793290. doi: 10.3389/fendo.2022.793290 (PMC8960113; doi:10.3389/fendo.2022.793290)

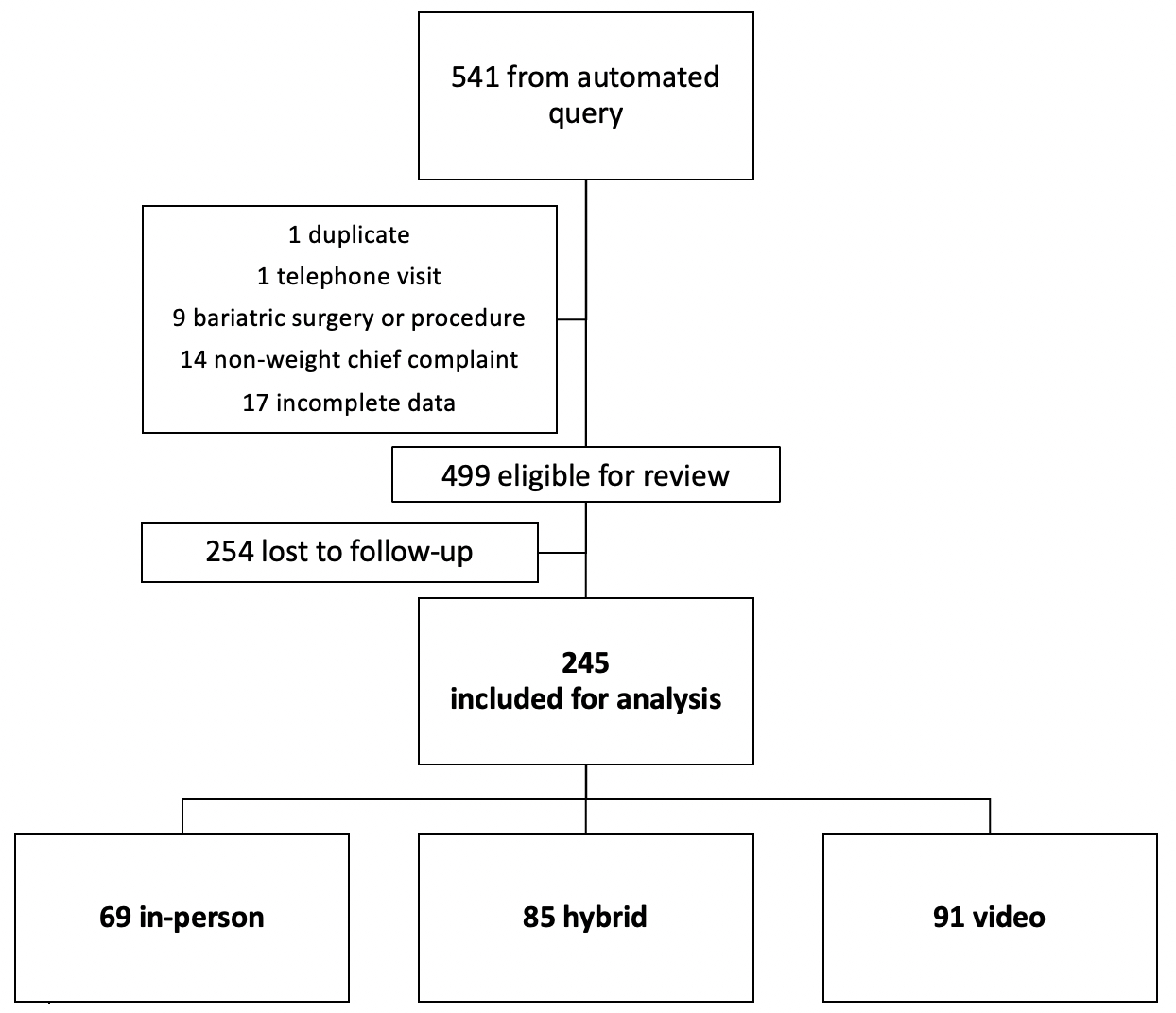

Supplement: Supplementary Figure 1 — Flowchart of patients included for analysis. [file Image_1.jpeg]
